# A protocol for a systematic review of the diagnostic accuracy of Loop-mediated-isothermal AMPlification (LAMP) in diagnosis of invasive meningococcal disease in children

**DOI:** 10.1186/s13643-018-0747-0

**Published:** 2018-06-15

**Authors:** Thomas Waterfield, Derek Fairley, Fiona Lynn, Bronagh Blackwood, Michael D. Shields

**Affiliations:** 1 Centre for Experimental Medicine, Wellcome Wolfson Institute of Experimental Medicine, Queen’s University Belfast, 97 Lisburn Road, Belfast, BT9 7AE UK; 2 Belfast Health and Social Care Trust, Belfast, UK

**Keywords:** Meningococcal, Meningitis, Sepsis, *Neisseria meningitidis*, Test accuracy, Meta-analysis

## Abstract

**Background:**

Meningococcal disease (MD) is notoriously difficult to diagnose in the early stages of the illness and presents similarly to many self-limiting viral infections. This mandates a cautious approach to diagnosis and initial management of suspected MD with many children receiving precautionary broad-spectrum intravenous antibiotics. Despite this approach, some children are still diagnosed late. In the last 10 years, there have been advances in nucleic acid amplification techniques, and there is now a rapid test that can detect meningococcal DNA in under 30 min. This Loop-mediated-isothermal AMPlification (LAMP) technology may make it possible to diagnose MD at initial presentation thereby greatly improving outcomes and minimising harms through unnecessary treatment. The aim of this systematic review is to determine the diagnostic accuracy of LAMP technology in cases of suspected MD.

The review has been registered with PROSPERO [CRD42017078026].

**Methods:**

To identify relevant studies, we will search MEDLINE, Embase, Web of Science, Scopus and The Cochrane Library. In additional, we will hand-search reference lists and grey literature including contacting the manufacturers of commercially available LAMP tests for MD for any unpublished data. Two reviewers will independently screen study eligibility and extract data. Methodological quality will be assessed, by two authors, according to the revised tool for the Quality Assessment of Diagnostic Accuracy Studies (QUADAS-2); any discrepancies will be resolved by a third author. The following test characteristics will be extracted into 2 × 2 tables for all included studies: true positives, false positives, true negatives, and false negatives. Study-specific estimates of sensitivity and specificity with 95% confidence intervals will be displayed in forest plots. To investigate heterogeneity, we will include covariates such as age, sample type, and study type into a bivariate random-effects model.

**Discussion:**

This review will help determine the diagnostic accuracy of LAMP technology in diagnosing MD from blood, CSF and throat swabs in children. The data will help to define where in the diagnostic pathway LAMP could be useful including potential as a point-of-care test for children at first presentation.

**Electronic supplementary material:**

The online version of this article (10.1186/s13643-018-0747-0) contains supplementary material, which is available to authorized users.

## Background

### Target condition being diagnosed

Despite successful vaccination programmes, meningococcal disease (MD) remains a leading infectious cause of septicaemia and death in children worldwide [1–5]. Early diagnosis of MD significantly improves outcomes with reduced morbidity and mortality [4, 5]. The challenge is, however, that during the prodrome invasive MD is indistinguishable from many self-limiting viral infections [4–6]. This invariably leads to a very cautious approach to the management of these children with many receiving parenteral antibiotics pending culture results [7]. Despite this cautious approach, children are still being diagnosed late due to the difficulties in identifying those children who are infected with MD from those who have a simple viral illness [4, 7].

Over the years, a number of studies have explored the value of widely available biomarkers including the use of CRP, Procalcitonin and white cell counts in the initial diagnosis of possible MD [8–12]. Whilst these tests have value, none of them have the necessary diagnostic accuracy to allow them to be used as rule out tests at presentation [8–12].

Loop-mediated-isothermal AMPlification (LAMP) for MD is a rapid form of PCR that targets the ctrA gene sequence. The ctrA gene sequence is genetically conserved across all pathogenic (capsular) stains of the bacterium *Neisseria meningitidis* that is responsible for MD [13]. This technique is faster than traditional PCR techniques and requires much simpler equipment [13–17]. It is possible that LAMP technology could be used as a rapid point of care test (POCT) for the early diagnosis of MD in children. This could be achieved through the rapid testing of blood samples or throat swab specimens in emergency departments, primary care facilities or pharmacies.

### Clinical pathway

It is very difficult to diagnose early meningococcal disease with current guidance recommending decision-making based on the clinical presentation and laboratory results [7, 12]. Unfortunately, no single biomarker, combination of biomarkers or clinical guideline has been found to be ideal [8–12, 18]. This has resulted in a very cautious approach resulting in the overtreatment of many children [8, 11, 12, 14]. Despite such a cautious approach, children are still being diagnosed late [12].

LAMP could potentially be used at two points within existing care pathways. Firstly at presentation to identify early invasive meningococcal disease in children who present with a minor illness [14]. Alternatively, LAMP could be used in place of the current gold standard (quantitative PCR or sterile site culture) to quickly confirm or exclude the diagnosis allowing for a more tailored treatment including early ambulation.

The development of LAMP technology for the diagnosis of early MD could therefore represent a significant breakthrough that could alter the care of thousands of children every year worldwide.

### Why perform this review?

This systematic review is required because there are a growing number of individual studies that have reported on the diagnostic accuracy of LAMP technology in diagnosing MD [13, 14, 19]. These studies have used a similar approach; LAMP directed at the conserved CtrA region of the *bacteria Neisseria meningitidis*, but in different populations using different specimens, i.e. blood, CSF and throat swabs [13, 14, 19]. There are, to our knowledge, no existing systematic reviews. This review may help researchers and policymakers identify the most suitable sample (blood, CSF, throat swab), and it may help to determine the role of LAMP within the existing diagnostic pathway.

### Objectives

The objective of this systematic review is to determine the diagnostic accuracy of LAMP technology in the diagnosis of invasive meningococcal disease in children (< 18 years of age).

## Methods/design

We will perform a literature search for relevant studies and then screen and select studies for inclusion against eligibility criteria. Data extraction will be performed in duplicate on the selected studies with meta-analysis and report writing.

We will adhere to standards of the Preferred Reporting Items for Systematic Reviews and Meta-Analyses (PRISMA) in reporting the findings of this review [20]. The content of this protocol follows the Preferred Reporting Items for Systematic Review and Meta-Analysis Protocols (PRISMA-P) recommendations [21]. (Please see the Additional file 1 for the completed PRISMA-P checklist.) This review is registered with the International Prospective Register of Systematic Reviews (PROSPERO) [22]. The registration number is [CRD42017078026].

### Inclusion criteria

Table 1 below outlines the inclusion criteria for this review. These criteria are discussed in more detail below.

**Table 1:** Eligibility criteria

Study characteristics	Inclusion criteria
Population	Children < 18 years of age with suspected meningococcal disease
Index tests	Loop-mediated-isothermal AMPlification for *Neisseria meningitidis*
Reference test	Quantitative PCR and/or culture of sterile site (blood and/or CSF) specimens
Outcomes	True and false positives, true and false negatives
Study designs	All prospective, retrospective and randomised control studies that report measures of diagnostic accuracy of Loop-mediated-isothermal AMPlification for *Neisseria meningitidis*

#### Types of studies

All prospective, retrospective and RCT studies that assess the performance of LAMP in assessing children (< 18 years of age) with potential invasive meningococcal disease will be included. There are no language restrictions.

#### Participants

The participants were children (< 18 years of age) with signs or symptoms of invasive meningococcal disease.

#### Index tests

The index test being investigated is the LAMP test for meningococcal DNA. For the purpose of this protocol, this is further defined as LAMP testing specific to the ctrA gene of *Neisseria meningitidis*. Index testing can be performed using blood, cerebrospinal fluid and throat swabs. Commercially and non-commercially available tests will be considered.

#### Target conditions

Meningococcal infection (invasive meningococcal disease) is the target condition.

#### Reference standards

The reference standard used to confirm the presence of the target condition in this study is quantitative PCR to detect *Neissseria meningitidis* DNA in a sterile site sample (normally blood or CSF). A positive blood or cerebrospinal bacterial culture of *Neissseria meningitidis* will also be used. No other reference standard will be accepted.

### Exclusion criteria

Studies that only assess carriage rates in healthy children will be excluded.

### Search methods for identification of studies

#### Electronic search strategy

An electronic search strategy has been developed in collaboration with the Queen’s University Belfast Medical Librarian (RF). To identify all prospective, retrospective and RCTs, we will search MEDLINE, Embase, Web of Science, Scopus and The Cochrane Library inclusive of Cochrane Controlled Trials Register. An example Medline search strategy is attached as Additional file 2. There are no language restrictions for this review.

#### Searching other resources

In addition, we will hand-search reference lists of relevant articles. A targeted grey literature search will include contacting the manufacturers of commercially available LAMP test for meningococcal disease and a search of conference abstracts.

### Data collection

#### Selection of studies

Two reviewers (TW, MDS) will independently screen the study eligibility and extract data. Screening will be a two-step process with initial title/abstract screening followed by full-text screening. Disagreements among reviewers will be resolved through consensus or third-party reviewer (DF). Reports that are duplicates or co-publications of studies will be identified. Following full-text screening, a list of excluded studies with reasons for exclusion will be provided in an appendix of the final report. We will begin with screening published and unpublished records and select those that meet the inclusion/exclusion criteria. Our search of literature will involve both primary studies and systematic reviews. The latter will be used only to identify additional primary studies.

#### Data extraction and management

TW and MDS will develop a data extraction form, and this will be piloted initially to achieve a good level of agreement between the data extractors. The following data will be extracted in duplicate by TW and MDS:

- Study characteristics: author, year of publication, country, design, sample size, clinical setting, number studied, number of drop-outs with reason, and funding source.
- Population characteristics: inclusion/exclusion criteria and patient demographics such as age and gender.
- LAMP testing: timing of sampling, method of sampling (e.g. throat swab, blood or CSF), time to result and commercial availability of the test.
- Gold standard: Quantitative PCR (e.g.TaqMan® PCR) or sterile site bacterial culture (i.e. blood/CSF)
- Outcomes: From this 2 × 2 table, we will calculate true positives, false positives, true negatives, and false negatives.

#### Assessment of methodological quality

The risk of bias of each article will be evaluated independently by two investigators (TW, MDS) and reported according to the Quality Assessment of Diagnostic Accuracy Studies (QUADAS-2) tool [23]. The most likely bias will be spectrum bias with participants not representing the population of interest. Early MD is very difficult to diagnose with typically vague and non-specific symptoms. Depending on the population, rates of MD can vary from 1 to 33% [8–11, 14]. We are primarily interested in LAMP testing as an early test to identify those children who will go on to deteriorate rather than considering its use in very unwell child in whom treatment will be given irrespective of the test result. Studies that focus on very sick children or studies that have an especially high rate of MD may not reflect the target population for the routine use of this test in the future. A commentary on this potential source of bias will be included, and where possible, we will compare the performance of LAMP as a diagnostic test in both low and high prevalence populations—discussed below. Disagreements between the two investigators (TW, MDS) will be resolved by consensus or arbitration by a third party (DF).

An assessment of publication bias will not be performed. There is no evidence of publication bias in systematic reviews of diagnostic accuracy, and methods for detecting publication bias are unreliable when applied to diagnostic accuracy studies [24].

### Statistical analyses and evidence synthesis

We will present an overview of the available studies summarised in two tables. The first table will summarise the study designs, participants, index tests details, sample types and the reference standards. The second table will summarise the details on study quality relating to QUADAS-2.

LAMP test result data will be compared to the reference test. Data for 2 × 2 tables of index test against reference standard will be extracted from each study. The true positive, true negative, false positive and false negative rate will be recorded. If these data are not provided, they will be calculated from raw data wherever possible. A summary table of evidence will be produced, and individual studies represented using forest plots displaying the sensitivity and specificity values of the LAMP test with 95% confidence intervals.

Inferred statistics: LAMP testing is binary with either a positive or negative result. There is no cutoff and the bacteria are either present or not. In the meta-analysis, we will therefore use a bivariate random-effects model with covariates such as age, sample type, disease incidence, variations in index tests and study type included to report summary statistics of sensitivity and specificity. This approach is recommended by the Cochrane collaboration and is best suited for the meta-analysis of diagnostic tests where there is no cutoff or the cutoff is very similar between studies [24]. All analyses will be performed in duplicate by TW and MDS using SPSS 23 and STATA.

In addition to the covariates included in the bivariate random-effects model already discussed, we will also perform subgroup analyses on the following groups:

- Infants (less than 1 year of age)
- Pre-school children (less than 4 years)
- School-age children (4–11 years)
- Adolescents (11–18 years)
- Sample type (throat swab, blood, CSF)
- High disease prevalence
- Low disease prevalence

### Investigations of heterogeneity

We will investigate the heterogeneity by incorporating covariates into the random-effects models as discussed above.

## Discussion

Meningococcal disease is a notoriously difficult disease to diagnose in the early stages, and as such many children are treated with precautionary intravenous broad-spectrum antibiotics. Despite this approach, children are still being diagnosed late with MD. Advances in nucleic amplification technology mean that it is now possible to perform LAMP testing with results being available in under 30 min. What we do not know, however, is how these tests can be used by clinicians in real-world situations. What is the diagnostic accuracy of LAMP testing for MD and how is affected by sample type and age of the child?

By answering these questions, we will have a better understanding of where LAMP testing could fit into the current clinical pathway for the diagnosis of MD. In particular, could it be used as a rapid point of care test to diagnose early MD, or could it have role in rapidly confirming diagnosis after the administration of antibiotics? This information will be of value to policy planners and researchers in determining where in the diagnostic pathway for MD the LAMP test could be trialled.

## Additional files

Additional file 1: PRISMA-P Checklist. (DOCX 33 kb)

Additional file 2: Example search strategy. (DOCX 99 kb)

## Abbreviations

CRP: C-reactive protein
CSF: Cerebral spinal fluid
DNA: Deoxyribonucleic acid
LAMP: Loop-mediated-isothermal AMPlification
MD: Meningococcal disease
NPV: Negative predictive value
PCR: Polymerase chain reaction
PCT: Procalcitonin
POCT: Point of care testing
PPV: Positive predictive value
RCT: Randomised control trial
