# Supplementary material for: A protocol for a systematic review of the diagnostic accuracy of Loop-mediated-isothermal AMPlification (LAMP) in diagnosis of invasive meningococcal disease in children
Source: Syst Rev. 2018 Jun 15;7:86. doi: 10.1186/s13643-018-0747-0 (PMC6003145; doi:10.1186/s13643-018-0747-0)
Supplement: Supplementary file 2 — Example search strategy. (DOCX 99 kb) [file 13643_2018_747_MOESM2_ESM.docx]

Medline Search Strategy:

Database(s): **Ovid MEDLINE(R) ALL**1946 to October 24, 2017 
Search Strategy:

| **#** | **Searches** | **Results** |
| --- | --- | --- |
| 1 | meningococcal infections/ or meningitis, meningococcal/ | 10614 |
| 2 | neisseria meningitidis/ or neisseria meningitidis, serogroup a/ or neisseria meningitidis, serogroup b/ or neisseria meningitidis, serogroup c/ or neisseria meningitidis, serogroup w-135/ or neisseria meningitidis, serogroup y/ | 9259 |
| 3 | "meningococcal disease*".mp. [mp=title, abstract, original title, name of substance word, subject heading word, keyword heading word, protocol supplementary concept word, rare disease supplementary concept word, unique identifier, synonyms] | 3691 |
| 4 | "meningococcal sepsis*".mp. [mp=title, abstract, original title, name of substance word, subject heading word, keyword heading word, protocol supplementary concept word, rare disease supplementary concept word, unique identifier, synonyms] | 361 |
| 5 | "invasive meningococcal".mp. [mp=title, abstract, original title, name of substance word, subject heading word, keyword heading word, protocol supplementary concept word, rare disease supplementary concept word, unique identifier, synonyms] | 826 |
| 6 | 1 or 2 or 3 or 4 or 5 | 15489 |
| 7 | "loop mediated isotherm*".mp. [mp=title, abstract, original title, name of substance word, subject heading word, keyword heading word, protocol supplementary concept word, rare disease supplementary concept word, unique identifier, synonyms] | 2033 |
| 8 | LAMP*.mp. [mp=title, abstract, original title, name of substance word, subject heading word, keyword heading word, protocol supplementary concept word, rare disease supplementary concept word, unique identifier, synonyms] | 28345 |
| 9 | 7 or 8 | 28532 |
| 10 | 6 and 9 | 16 |
